# Implementing Systematic Screening and Structured Care for Distressed Callers Using Cancer Council’s Telephone Services: Protocol for a Randomized Stepped-Wedge Trial

**DOI:** 10.2196/12473

**Published:** 2019-05-16

**Authors:** Elizabeth A Fradgley, Anna Boltong, Lorna O'Brien, Allison W Boyes, Katherine Lane, Annette Beattie, Tara Clinton-McHarg, Paul B Jacobsen, Christopher Doran, Daniel Barker, Della Roach, Jo Taylor, Christine L Paul

**Affiliations:** 1 Priority Research Centre for Cancer Research Innovation and Translation University of Newcastle Callaghan Australia; 2 Priority Research Centre for Health Behaviour University of Newcastle Callaghan Australia; 3 School of Medicine and Public Health University of Newcastle Callaghan Australia; 4 Strategy and Support Cancer Council Victoria Melbourne Australia; 5 St John of God Hospital Richmond Australia; 6 Hunter Medical Research Institute Callaghan Australia; 7 Cancer Information and Support Services Cancer Council Victoria Melbourne Australia; 8 Cancer Information and Support Services Cancer Council NSW Woolloomooloo Australia; 9 School of Psychology University of Newcastle Callaghan Australia; 10 Healthcare Delivery Research Program National Cancer Institute Bethesda, MD United States; 11 Centre for Indigenous Health Equity Research School of Health, Medical and Applied Sciences University of Central Queensland Brisbane Australia

**Keywords:** psycho-oncology, cancer, psychological stress, community health services, telephone hotlines

## Abstract

**Background:**

Structured distress management, comprised a 2-stage screening and referral model, can direct supportive care resources toward individuals who are most likely to benefit. This structured approach has yet to be trialed in Australian community-based services such as Cancer Council New South Wales (NSW) and Victoria Cancer Information and Support (CIS) 13 11 20 lines who care for a large community of cancer patients and caregivers.

**Objective:**

The aim of this study was to evaluate the effectiveness of structured screening and referral in (1) increasing the proportion of distressed CIS callers who accept supportive care referrals and (2) reducing distress levels at 6-month follow-up.

**Methods:**

In this stepped-wedge trial, Cancer Council NSW and Victoria CIS consultants are randomized to deliver structured care during inbound 13 11 20 calls in accordance with 3 intervention periods. Eligible callers are patients or caregivers who score 4 or more on the Distress Thermometer; NSW or Victorian residents; aged 18 years or older; and English proficient. Study data are collected via computer-assisted telephone interviews (CATIs) at 3- and 6-month follow-up and CIS record audit. CATIs include demographic and service use items and the General Health Questionnaire (GHQ-28) to assess distress. An economic analysis of the structured care model will be completed.

**Results:**

The structured care model was developed by guideline review and identification of service characteristics to guide mapping decisions; place-card methodology; and clinical vignettes with think-aloud methodology to confirm referral appropriateness. The model includes an additional screening tool (Patient Health Questionnaire-4) and a referral model with 16-20 CIS services. Descriptive statistics will be used to assess referral uptake rates. Differences between GHQ-28 scores for structured and usual care callers will be tested using a generalized linear mixed model with fixed effects for intervention and each time period. The trial will recruit 1512 callers. The sample size will provide the study with approximately 80% power to detect a difference of 0.3 SD in the mean score of the GHQ-28 at an alpha level of .05 and assuming an intra-cluster correlation of .04. A random sample of recorded calls will be reviewed to assess intervention fidelity and contamination. To date, 1835 distressed callers have been invited to participate with 60.71% (1114/1835) enrolled in the study. A total of 692 participants have completed 6-month CATIs. Recruitment is anticipated to end in late 2019.

**Conclusions:**

This trial is among the first to rigorously test the outcomes of a community-based structured approach to distress management. The model is evidence-informed, practice-ready, and trialed in a real-world setting. The study outcomes will advance the understanding of distress management internationally for both patients and caregivers.

**Trial Registration:**

Australian New Zealand Clinical Trial Registry ACTRN12617000352303; https://www.anzctr.org.au/Trial/Registration/TrialReview.aspx?id=372105&isReview=true (Archived by WebCite on http://www.webcitation.org/78AW0Ba09)

**International Registered Report Identifier (IRRID):**

DERR1-10.2196/12473

## Introduction

### Identifying and Responding to Cancer-Related Distress

Cancer-related distress is defined as a multifactorial unpleasant emotional experience of a psychological, social, or spiritual nature which may interfere with the ability to cope effectively with the disease, its symptoms, and treatment [1]. A recent literature review of symptom prevalence suggests distress is experienced by approximately 40% of cancer patients [2]. Australian longitudinal data have also demonstrated that up to 31% of caregivers experience borderline or clinical anxiety [3].

Distress and psychological morbidity among people affected by cancer are associated with decreased social functioning, more intense physical symptoms, cognitive impairment, poor adherence to treatment, and reduced length of life [4,5]. Internationally, there are distress screening and management guidelines available; Australian examples include Cancer Australia’s Clinical Guidance for Responding to Suffering in Adults with Cancer [6] and the Clinical pathway for the screening, assessment and management of anxiety and depression in adult cancer patients [7]. Guidelines recommend using a brief distress screening tool, such as the distress thermometer (DT) or Edmonton Symptom Assessment Scale.

Evidence from clinical settings suggests timely identification when paired with structured management of psychological distress can improve medical management and reduce distress [8-10]. This evidence must be cautiously interpreted as other studies demonstrate inconsistent or minimal improvement in patient outcomes following screening [11]. The debate surrounding the utility of distress screening is ongoing and complex [12,13]. However, any program that involves tokenistic distress screening without a feasible referral pathway is unlikely to influence patient outcomes or experiences. Furthermore, screening models may be improved by focusing on patients’ adaptive or maladaptive reactions to their distress in addition to severity [14]. This trial will contribute further data to the debate by involving new settings in which structured distress management has not yet been trialed.

### Incorporating and Evaluating Distress Screening Practices in Telephone-Based Services

The International Psycho-Oncology Society emphasized that “Distress should be recognized, monitored, documented and treated promptly at all stages of disease and *in all settings*.” [15]. One setting in which structured care might be implemented routinely is the community-based Australian Cancer Council Cancer Information and Support (CIS) telephone service. The CIS model of care operates in numerous countries [16] including the United Kingdom [17] and Australia [18]. Under the Australian CIS model, health professionals provide emotional, practical, and informational telephone support to both patients and caregivers [18]. Strengths of the model include its ability to assist individuals who are unable to receive traditional face-to-face supportive care owing to geographical isolation or poor physical health [17]. In 2017, the Australian CIS 13 11 20 telephone service received 46,000 calls nationally [19], of which, the New South Wales (NSW) CIS received 12,225 calls [20] and Victorian CIS service received 11,429 calls [21].

In total, 2 Australian studies established the acceptability and feasibility of implementing distress screening and tiered care in the CIS context [22,23]. These exploratory studies revealed that, despite screening acceptability, a low proportion of callers take up the referrals that are offered. Poor referral uptake (approximately 20% to 25%) has also been reported in hospital-based services [24,25]. As additional psychosocial care is associated with improved emotional well-being and quality of life, it is critical to maximize the proportion of distressed patients and caregivers acting upon these referrals [26].

This stepped-wedge trial rigorously tests the uptake, likely impact, and costs of a structured care approach to distress screening and management across the NSW and Victorian CIS telephone services. The trial compares the effectiveness of a distress screening model using the DT only (ie, usual care) against a 2-staged distress screening model incorporating the DT, Patient Health Questionnaire (PHQ-4), and a referral model (ie, structured care model). Effectiveness of the structured care model is gauged by distressed callers’ referral uptake rates and 6-month distress levels measured by the General Health Questionnaire (GHQ-28). Usual and structured care are delivered by CIS consultants during inbound calls from distressed cancer patients and caregivers.

This trial was prospectively registered with the Australian New Zealand Clinical Trials Registry (ACTRN12617000352303) on March 8, 2017.

### Aims

To identify the following:

1. The proportion of distressed people affected by cancer who call the CIS service and take up an offer of referral under the structured care model.
2. Whether the structured care model reduces the level of distress at 6-month follow-up among people affected by cancer when compared with usual care.
3. The relative costs and benefits of structured versus usual care models at 6-month follow-up from the service provider perspective.

### Hypotheses

At 6-month follow-up:

1. There will be at least a 20% increase in the proportion of distressed callers who are offered a referral under the structured care model and accept the offer.
2. Distressed callers who receive structured care referrals will report lower scores on the GHQ-28 (0.3 SD lower) at 6 months when compared with those who receive usual care.
3. Structured care will incur higher service delivery costs per distressed caller than usual care. These higher costs will be considered appropriate by the service provider and consumers to reduce caller distress.

## Methods

### Study Design

The stepped-wedge trial is conducted with the Cancer Council NSW and Victoria CIS 13 11 20 line. The structured care model is sequentially rolled out with CIS consultants randomly allocated to transition to structured care over 3 intervention periods (Table 1). There is a 1-month transition period between each intervention period. Transition periods are included in reporting guidelines for stepped-wedge trials [27]; the transition periods allow for consultants to trial the new structured care call content (PHQ-4 and referral model), discuss and iteratively refine new content with other structured care consultants and receive feedback from the research team.

On the basis of previous CIS call volumes, the trial is approximately 24 months with the opportunity to adjust timeframes in accordance with CIS caller recruitment rates and other internal requirements (eg, implementation of a new electronic medical record system). The study is reported according to SPIRIT recommendations [28].

**Table 1:** Consultant allocation to intervention periods with caller sample size.

Consultant^a^	Intervention period (n=504)				
	1	Transition	2	Transition	3
A	Usual care	→	Structured care	—^b^	Structured care
B	Usual care	→	Structured care	—^b^	Structured care
C	Usual care	→	Structured care	—^b^	Structured care
D	Usual care	→	Structured care	—^b^	Structured care
E	Usual care	—^b^	Usual care	→	Structured care
F	Usual care	—^b^	Usual care	→	Structured care
G	Usual care	—^b^	Usual care	→	Structured care
H	Usual care	—^b^	Usual care	→	Structured care

^a^Each Cancer Information and Support (CIS) service has at least 4 consultants participating in the study.

^b^Not applicable. No transition required.

### Caller Eligibility and Recruitment

#### Caller Eligibility

Eligible participants are inbound callers to the 13 11 20 CIS who reside in NSW or Victoria; are aged 18 years or older; have been diagnosed with cancer or support someone with cancer; have DT scores of 4 or more; and have provided consent to telephone follow-up. A meta-analysis of use of the DT with patients diagnosed with cancer found a sensitivity of 81% and specificity of 72% to detect distress using a cut-off score of 4 [29].

#### Caller Recruitment

**Figure 1:**
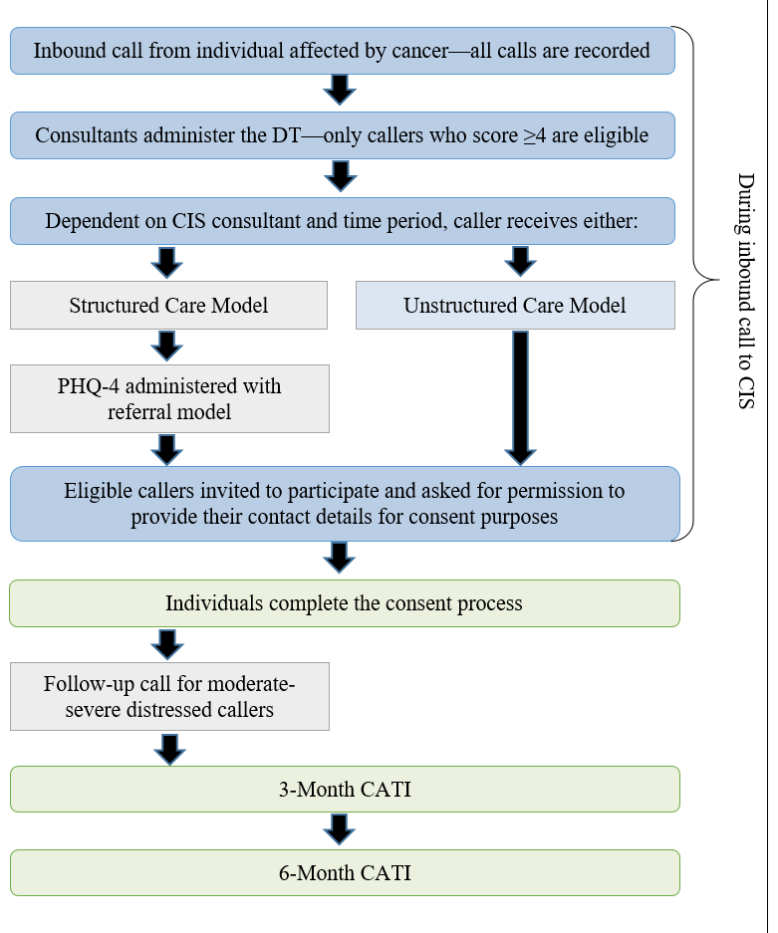
Brief description of call content, recruitment process, and computer assisted telephone interviews (CATIs) time points.

At the end of the inbound call, eligible individuals are invited by consultants to participate and are asked for permission to pass their contact details to the researchers. After receiving the contact details, the research team post study packages to potential participants with 3 consent options: written consent via return post; electronic consent via online form or email; or verbal consent with the research team. Individuals who do not return a consent form within 10 days are contacted by telephone. Basic data on callers who either declined to provide their contact or declined participation after receiving a study package will be analyzed to ascertain any consent bias. See Figure 1 for a brief overview of caller recruitment, call content, and data collection time points.

### Consultant Training and Randomization

#### Consultant Qualifications

CIS consultants are qualified oncology or psychosocial professionals such as specialist oncology nurses, psychologists or counsellors, and social workers. To account for the multidisciplinary backgrounds of CIS consultants, each consultant receives training in therapeutic communication skills and routinely participate in clinical supervision and professional development workshops. As part of the training, new consultants also complete clinical shadowing and receive simulated calls as a quality assurance exercise.

#### Intervention Training

To participate in this study, consultants participated in 2 3-hour face-to-face group workshops: (1) usual care training focused on administering the DT and recruiting callers and (2) structured care training focused on integrating the PHQ-4 and referral model into calls. Each interactive workshop is followed by a group videoconference to troubleshoot issues and reinforce new behaviors. Booster training is provided at 3- to 6-month intervals depending on the need.

#### Consultant Randomization

Consultants are randomized to deliver either usual care or structured care during inbound calls with all consultants delivering structured care (ie, PHQ-4 with the referral model) at study completion (Table 1). A randomization table was created by computer software (ie, computerized sequence generation) and using stratified block randomization by state. Owing to the nature of the intervention, consultants are not blinded to the allocation and callers are not informed of consultants’ allocation.

#### Unstructured Care Calls (Usual Care Condition)

All CIS inbound calls begin with an opening such as “How can I help you today?” The conversation is caller-directed, in that the consultant seeks to understand the reason for the call and establish a level of rapport with the caller using responsive listening and expressions of empathy. Although the ensuing care is not formally structured, all consultants use the same client record management (CRM) system which provides a common framework and resources to guide the scope and progress of calls. Inbound calls can last from 10 to 45 minutes.

Under usual care, all callers are screened using the DT. Callers may be offered any of the following internal CIS services: (1) online peer-based support and information; (2) face-to-face support groups where people affected by cancer support each other; (3) one-to-one telephone support from a person who has recovered from a similar experience; (4) telephone support group meetings of 3 to 7 members and qualified facilitators twice a month; (5) referral to social work, legal, financial, or transport assistance; (6) cancer survivor programs such as a personalized diet and exercise programs; (7) information resources; or (8) referral to a psychologist coordinated via CIS. These referrals can be introduced at any point throughout the call.

Callers who speak to unstructured care consultants are not denied any services, and callers may request specific services. The usual care group is not assessed for service suitability in the systematic and structured fashion which will be the case for the intervention group.

#### Structured Care Calls (Intervention Condition)

A structured care call begins with the same openings as in usual care, is caller-directed, and callers are also screened with the DT. To deliver structured care, consultants administer an additional screening tool and apply a referral model based on screening results (Multimedia Appendix 1). There is no specific time point at which the consultants must introduce additional screening tools and referrals during the call, so the discussion remains caller-directed and conversational. Similar to callers who speak to unstructured care consultants, those who receive structured care are not denied any services and callers may request specific services.

##### Structured Care Screening Tools

The first screening stage is conducted using the DT. Those who score 4 or more subsequently complete the PHQ-4. The PHQ-4 has been used in cancer samples previously and has been shown to be accurate and reliable [30]. The PHQ-4 is brief and well-suited to the conversational style of CIS consultations. To report severity of distress, the PHQ-4 consists of 2 items regarding depressed mood (PHQ-2) and 2 items from the General Anxiety Disorder [(GAD-2)] tool relating to anxious mood. Using a Likert scale, respondents indicate how often they experienced the symptom in the previous 2 weeks. PHQ-4 scores range from 0 to 12 with higher scores indicating greater distress severity. The PHQ-4 score will be used to determine which services are most appropriate for each caller based on the structured care referral model.

##### Structured Care Referral Model

To develop a referral model that is evidence-informed and a pragmatic combination of distress screening, tiered care, and stepped care, the team held a 2-day workshop to map internal CIS services to PHQ-4 distress scores. The iterative model development process included group review of existing guidelines; identification of service characteristics to guide mapping decisions (eg, frequency and health professional delivering the intervention); place-card methodology to arrange services by PHQ-4 scores; and application of clinical vignettes with think-aloud methodology to confirm appropriateness of referral decisions. The referral model was further refined following a pilot test with approximately 40 distressed callers. Examples of changes to the model include reformatting to a pyramid shape with 3 tiers; additional detail on timing of an outbound follow-up call; and description of universal care options such as CIS information brochures.

The final referral model includes 16 to 20 CIS services across 3 levels of distress and is state-specific, given different service availability and branding (Multimedia Appendix 1). The referral model also includes an additional follow-up call to repeat screening and further support those individuals with elevated or unchanged distress scores. If accepted, the timing of the call is determined by caller preferences to accommodate pivotal moments in the cancer journey such as treatment commencement.

#### Intervention Fidelity and Contamination

All calls to the Cancer Council are recorded as a part of standard operating procedures and the research team will review a random sample of recorded phone calls. Using the CIS CRM Systems, the research team will audit the proportion of calls in which the PHQ-4 scores were recorded to determine fidelity. Contamination between the 2 groups, for example, use of the PHQ-4 items, will also be evaluated by auditing usual care calls in addition to structured care calls for fidelity. Contamination between usual care consultants and structured care consultants is minimized by strategic design of the CRM system so that the PHQ-4 is not easily viewable by usual care consultants and separated training sessions; and, as a part of training, consultants were informed about the trial design and the need to reduce contamination.

### Study Outcomes

Study outcomes are assessed through 6-month computer assisted telephone interviews (CATIs) with participants; the CIS CRM Systems; and review of audio recordings. The data collection time periods were designed to increase patient recall of referrals in 2 shorter 3-month periods. CATIs are an appropriate method of data collection from participants recruited through a telephone-based support service.

Referral uptake is the primary outcome and is measured as the proportion of participants who report being provided with a referral and report at 6-month follow-up that an action has been taken to progress the referral (ie, an appointment or telephone interaction). Referral uptake was selected as the primary outcome as it can reflect the appropriateness of the type of referral offered to callers under the structured care model. The hypothesized increase of 20% in referral uptake was selected based on internal CIS data in the absence of comparable studies in telephone-based supportive care service uptake. The study-specific questions are tailored to the CIS services; questions were pilot-tested with a consumer advisory panel and reviewed after the first 20 participants.

Distress is measured by the GHQ-28 [31] that is a widely used self-report measure of general psychological distress. The measure uses 28 items to assess perception of health in terms of ability to play a useful part in life; make decisions; overcome difficulties; enjoy normal activities; face problems; and feel confident, worthwhile, and happy [31]. The GHQ-28 has excellent internal consistency, diagnostic accuracy, and test-retest reliability [32,33]. The measure has been used in the Australian community [34] and with patients with cancer [35].

#### Moderators

Sociodemographic and disease-related characteristics include cancer type, stage of disease, age, gender, postcode, marital status, education, health care card recipient, private health insurance, household income, previous psychological treatment, and/or morbidity and other assistance received since study enrolment. Caller type (patient or caregiver), DT score at inbound CIS call, and reason for contacting the CIS (eg, information, emotional, or practical support) will also be accounted for in study analyses. Subgroup analyses will be conducted for caregivers. Basic information regarding the consultants, such as the number of years at the CIS, will also be incorporated into analyses.

The Health Education Impact Questionnaire (HeiQ) is a 42-item tool for assessing the efficacy and impact of health education and self-management programs for people with chronic diseases [36]. The HeiQ has demonstrated reliability and validity, including with oncology samples, and has been used previously by Cancer Councils to evaluate programs [37,38].

#### Acceptability

The consultants’ experience in using the structured care model, and the perceived value of this approach, will be explored in qualitative interviews at conclusion of the study. The semistructured interviews will include questions about the value of the additional questions and referral model at eliciting and managing emotional distress and the impact of these additions in building rapport and maintaining a caller-directed approach. The interview will be recorded, partially transcribed, and analyzed according to content analysis facilitated through NVivo qualitative data analysis software (QSR International Pty Ltd. Version 10, 2014). The Consolidated Criteria for Reporting Qualitative Research will be used [39].

#### Costs

The length and number of calls made or received by participants is automatically recorded. Standard hourly rates for consultants will be used to calculate the cost of service delivery. Service provider costs owing to the uptake of referrals in both groups are tracked through the Cancer Council CRM, and standard hourly costs will be applied to provide a full assessment of the cost implications for each of the care models. Standard hourly costs will be derived directly from CIS CRM data.

Participant outcome data and service provider cost data will be submitted to a series of discussion sessions involving consumers and relevant service leads at conclusion of the study. These sessions will explore whether the identified consumer benefits are perceived to be commensurate with the additional service provider costs incurred. The discussion sessions will be guided by the Nominal Group Technique [40]: (1) Service leads will receive a prediscussion report with information such as cost per caller screened under usual and structured care, odds of referral uptake, and average change in distress scores; (2) The roundtable discussion will be led by a group facilitator who will ask for individual feedback recorded in a *round-robin* format; (3) The group will then collectively discuss the feedback until consensus is reached on the cost and benefits of intervention. This discussion will be audio-recorded; and 4) Participants will have the opportunity to provide further comment directly to the research team.

#### Statistical Methods and Sample Size

Descriptive statistics will assess referral uptake rates. Differences between GHQ-28 scores for the structured and usual care groups will be tested using a generalized linear mixed model with fixed effects for the intervention and each time period after baseline. To account for the fact that outcomes are measured at the participant level while randomization is at the consultant level, a normally distributed random intercept for consultants will be included in the model. The parameter of interest from these models will be the estimated coefficient for the intervention term. On the basis of 8 consultants participating, the trial aims to recruit a total of 1512 distressed callers across the 3 steps. The sample size provides the study with approximately 80% power to detect a difference of 0.3 SD in the mean score of the GHQ-28 at an alpha level of .05 and assuming an intracluster correlation of .04. An effect size of .3 was selected based on previous trials of psychoeducation and telephone-administered interventions with patients with cancer [41].

#### Ethics

The study has been approved by the University of Newcastle Human Research Ethics Committee (NHMRC Committee Code: EC00144; Reference No. H-2016-0180); the Cancer Council of NSW (NHMRC Committee Code: EC00345; Reference No. 304); and the Cancer Council Victoria (NHMRC Committee Code: EC00203; Reference No. 1605).

## Results

### Progress

As of April 2019, 1835 eligible CIS callers have been invited to participate in the study by CIS consultants. A total of 1114 (60.70%, 1114/1835) individuals consented to participate; 372 (20.27%, 372/1835) declined to participate; and 180 (9.81%, 180/1835) did not respond to the invitation to participate after postal and telephone reminders. The consent status of the remaining 79 individuals is not yet known, and the individuals are currently receiving follow-up reminders through phone and email prompts.

Of the 1114 consenting participants, 692 have now completed the 6-month CATI. From the start of the study, 182 enrolled participants did not complete their 6-month CATI—this represents a lost-to-follow-up of 20.8% (182/874). Just over half of the current sample (56.01%, 624/1114) are patients. The remaining 44.00% (490/1114) of participants are individuals supporting someone with a cancer diagnosis, in remission or bereaved.

### Timeline

The trial entered the final intervention stage in April 2019, with all participating CIS consultants now delivering structured care. Study recruitment will continue for 6 months after this transition, with a further 6 months required to complete follow-up assessments. As such, trial outcome data are anticipated to be available in early 2020.

## Discussion

### Evidence-Informed Distress Screening by Telephone-Based Services Can Fill an Important Gap in Supportive Care

Distress among patients with cancer and caregivers is recognized as an important and challenging issue and is yet to be managed in a widespread, consistent, and effective fashion [11]. The Cancer Council CIS services hold a unique coordination role within community-based cancer care by facilitating access to additional psychosocial support programs outside of hospital-based settings. The CIS services also have tremendous potential to support caregivers and survivors who will experience clinically significant distress [2,3]. Furthermore, the CIS can provide a safety net by identifying the many distressed patients with cancer who *fall-through the cracks* in Australian cancer services [42,43]. For example, a previous Australian study suggested that a third of cancer services do not routinely screen outpatients for distress [42].

### Study Contribution to the Literature on Distress Screening and Management

The intention for any community-based telephone counselling service, such as the CIS services, to implement distress screening and various forms of structured care requires thorough evaluation of the benefits to callers. These benefits must be considered alongside the additional staff time and potential changes to the way staff interact with callers. For example, it is unknown if additional screening may affect the type and intensity of support offered to distressed callers, particularly as emotional well-being may not have been the motivating reason for contacting the service. A key strength of this study is the diverse sample of patients and caregivers recruited to mimic the large and heterogeneous community supported by the Cancer Council CIS services and other international telephone counselling services [16-18]. As evidence suggests that caregivers will experience distress levels similar to those of patients [3], the study will provide an invaluable opportunity to specifically report on the experience of distressed caregivers and their use of supportive care services.

As a critical factor for the translation of research into practice, this study has a strong focus on the resource implications of implementing a structured care model. The final phase of the proposed study involves facilitated discussion with service leaders and managers to examine the psychosocial outcome data alongside the cost data. This process will assist the end users in assessing the resource implications of implementing structured care, which will be important for the translation and sustainability of a structured care approach. The assessor comments have been provided in Multimedia Appendix 2.

This trial is the first to rigorously test the outcomes of a community-based (rather than clinical) structured approach to distress management. The model is evidence-informed, practice-ready, and trialed in a real-world setting. The outcomes of this trial will advance the understanding of distress management internationally. The proposed trial is also one of the first to deliver a harmonized multi-state intervention across state-based CIS borders. As the CIS service reported approximately 46,000 calls nationally in 2017, it is evident that an effective and consistent distress management model has tremendous potential to improve the psychosocial care for a large number of patients with cancer and caregivers.

## Appendix

Multimedia Appendix 1 Example of referral model.

Multimedia Appendix 2 Assessor comments as part of National Health and Medical Research Council peer-review process.

## Abbreviations

CATI: computer assisted telephone interview
CIS: Cancer Information and Support
CRM: client record management
DT: Distress Thermometer
GHQ: General Health Questionnaire
HeiQ: Health Education Impact Questionnaire
NHMRC: Newcastle Human Research Ethics Committee
NSW: New South Wales
PHQ: Patient Health Questionnaire
