# Supplementary figures and images for: Implementing Systematic Screening and Structured Care for Distressed Callers Using Cancer Council’s Telephone Services: Protocol for a Randomized Stepped-Wedge Trial
Source: JMIR Res Protoc. 2019 May 16;8(5):e12473. doi: 10.2196/12473 (PMC6542249; doi:10.2196/12473)

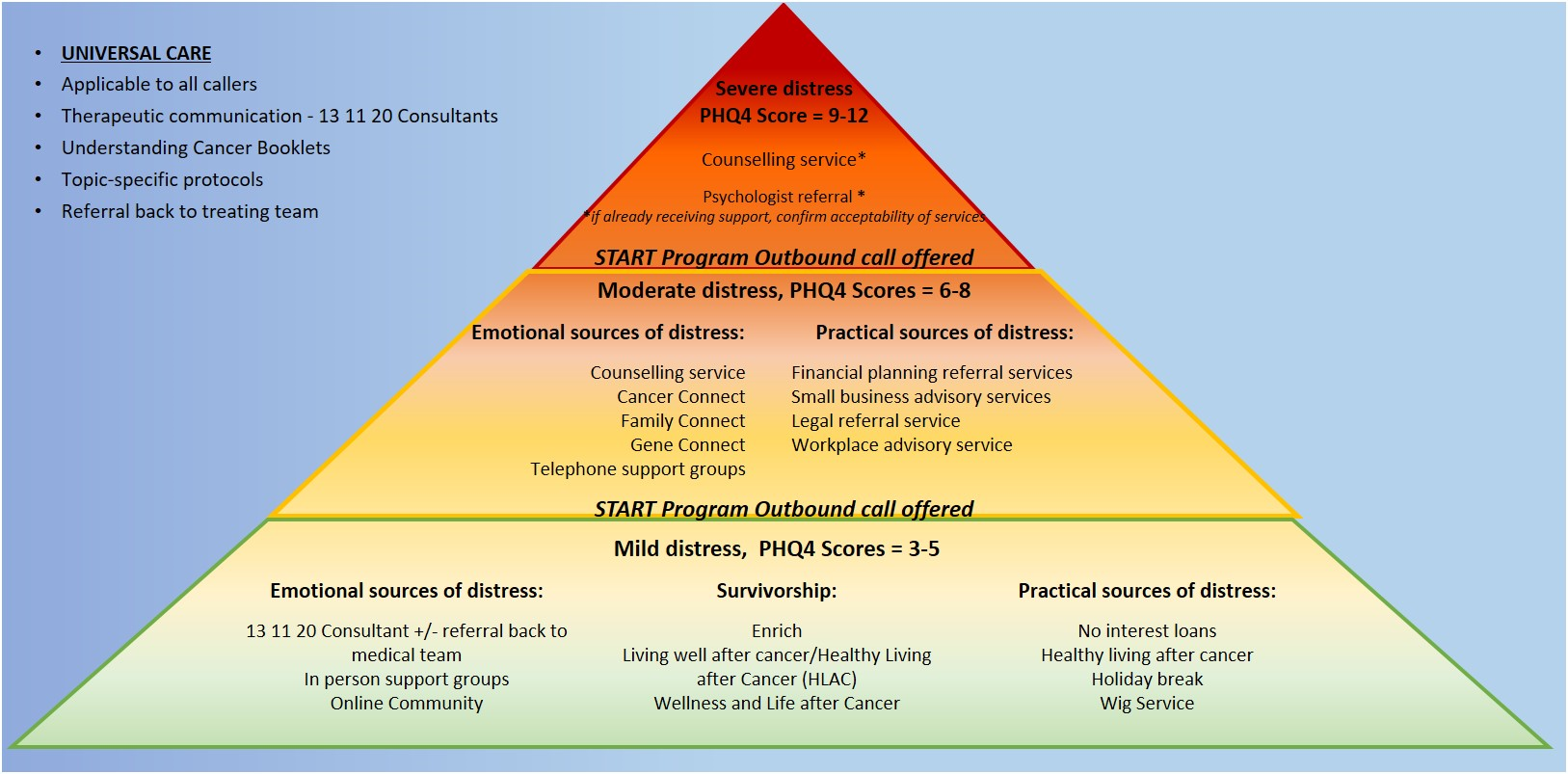

Supplement: Multimedia Appendix 1 [file resprot_v8i5e12473_app1.png]
