# Supplementary material for: Implementing Systematic Screening and Structured Care for Distressed Callers Using Cancer Council’s Telephone Services: Protocol for a Randomized Stepped-Wedge Trial
Source: JMIR Res Protoc. 2019 May 16;8(5):e12473. doi: 10.2196/12473 (PMC6542249; doi:10.2196/12473)
Supplement: Multimedia Appendix 2 [file resprot_v8i5e12473_app2.pdf]

GPO Box 1421 | Canberra ACT 2601
16 Marcus Clarke Street, Canberra City ACT 2600
T. 13 000 NHMRC (13 000 64672) or +61 2 6217 9000
F. +61 2 6217 9100
E. nhmrc@nhmrc.gov.au
ABN 88 601 010 284
www.nhmrc.gov.au

Attachment A: Project 2015 Assessor Report

**Application ID**	APP1108415
**CIA Name**	Associate Professor Christine Paul
**Application Title**	A randomised trial of systematic distress screening and structured care for callers using Cancer Councils' telephone support services

Assessor 1

01. Scientific Quality (Refer to the information available in the Research Proposal section of the Grant Proposal.
Assessors will:
- *Identify major and minor strengths and weaknesses in the proposed research plan.*
- *Consider the clarity and feasibility of the hypothesis or research objectives.*
- *Ask specific questions the Applicant can answer.*
- *Not provide a narrative or description of the proposed research.)*

well written and well structured proposal The need for economic assessment ewas mentioned/implied but was not further discussed or developed

Assessor 1

02. Significance of the expected outcomes AND/OR Innovation of the concept (Refer to the information available in the Research Proposal section of the Grant Proposal.
Assessors will:
- *Consider whether the proposed research has potential to increase knowledge about human health, disease diagnoses, or biology of agents that affect human health. Consideration should also be applied to the application of new ideas, procedures, technologies, programs or health policy settings to important topics that will impact on human health.*
- *Assess any new/novel transformative aims and cutting edge techniques which could result in a paradigm shift affecting current practices or approaches.*
- *Reflect upon whether the proposed research is likely to result in yielding highly influential publications.*
- *Evaluate potential interest from other external parties, for example other researchers or community groups, following the outcomes of the research.*
- *Ask associated specific questions the Applicant can answer.*
- *Not provide a narrative or description of the proposed research.)*

Clearly an important topic and the 'right' time to do the study; however would need further follow-up to assess whether distress and psychological risk and morbidity has been resolved

Assessor 1

03. Team quality and capability – relative to opportunity (Refer to the Team Quality and Capability section of the Grant proposal.
Assessors will:
- *Assess the track record of the CI team as a whole, relative to opportunity (Evaluate the support Junior Researchers (if applicable) have within the team), in context to the proposed research and time commitment of each CI.*

• *Consider all disclosed career disruptions and whether the referenced career disruption meets the NHMRC's definition (Section 6.1.1 of the NHMRC Funding Rules 2015) and if so, what the impact of the career disruption has had on the applicant's research achievements against all of the assessment criteria. Further advice pertaining to career disruptions can be found at Attachment H of the Project Grants Scheme Specific peer review guidelines.*
• *Evaluate clinical/teaching loads, output (publication quality and/or frequency) and contribution to discipline or field of expertise.*
• *Not consider journal impact factors or person centric metrics such as H-index. Citations are appropriate.)*

team has the appropriate skills and mix with high productivity. It also seems coherent and good to see involvement of service providers

<u>Assessor 1</u>
Budget Comments

budget requests seem reasonable

<u>Assessor 1</u>
Overall Comments

None provided.

<u>Assessor 2</u>
01. Scientific Quality (Refer to the information available in the Research Proposal section of the Grant Proposal. Assessors will:
• *Identify major and minor strengths and weaknesses in the proposed research plan.*
• *Consider the clarity and feasibility of the hypothesis or research objectives.*
• *Ask specific questions the Applicant can answer.*
• *Not provide a narrative or description of the proposed research.)*

This proposal addresses a defined need in cancer populations (i.e., psychological distress). There are two hypotheses (1. predicting lower distress in the Intervention group and 2. predicting that costs of the intervention will be appropriate in terms of improved client outcomes). Hypothesis 1 predicts a very small group difference, raising the question of the value of such an intervention. Clinically it is questionable whether a difference of 0.2 of a Standard Deviation would be meaningful. Hypothesis 2 lacks clarity as the terms "appropriate" and "improved client outcomes" are not well defined. Another concern is the lack of control of the content of Usual Care. It does not appear that the specific content of this condition will be monitored in the study (although the length of the phone consultations will be timed). As Usual Care is "unstructured" there is a possibility that one of the consultants could provide recommendations not dissimilar to that of the Intervention operators - there does not seem to be any mechanism outlined that would capture this situation. It is also possible that the longer time spent by consultants with Intervention participants may lead to differences in outcomes. Since sessions are being recorded it should be possible to consider time spent as a possible covariate. It is noted that the consultants will be trained and monitored to ensure protocol adherence, but it is not clear how can this be achieved with the unstructured usual care group. It is estimated that 1400 individuals will consent to be in the study, which is appropriate given the small effect size hypothesised, and appears feasible given that more than 15,000 individuals contact the Cancer Council's CIS services each year. However, it is not clear what percentage of these callers are likely to have a distress level of 4 or greater (a requirement for study entry). Some greater detail on these numbers would improve the case for this study being both feasible and representative.

<u>Assessor 2</u>
02. Significance of the expected outcomes AND/OR Innovation of the concept (Refer to the information available in the Research Proposal section of the Grant Proposal.
Assessors will:
• *Consider whether the proposed research has potential to increase knowledge about human health, disease diagnoses, or biology of agents that affect human health. Consideration should also be applied to the application of new ideas, procedures, technologies, programs or health policy settings to important topics that will impact on human health.*
• *Assess any new/novel transformative aims and cutting edge techniques which could result in a paradigm shift affecting current practices or approaches.*
• *Reflect upon whether the proposed research is likely to result in yielding highly influential publications.*
• *Evaluate potential interest from other external parties, for example other researchers or community groups, following the outcomes of the research.*
• *Ask associated specific questions the Applicant can answer.*
• *Not provide a narrative or description of the proposed research.)*

Since there is a move towards mandatory distress screening for callers to CIS services, this study has the potential to provide timely data regarding the most effective means by which to provide support and management of individuals experiencing heightened distress. This research will help inform best practice for community-based cancer support services, and may lead to international adoption due to links with CI Jacobsen (USA). One aspect that has been underplayed in this protocol is an argument why harmonisation of services across the nation is of value. This is alluded to but a stronger argument could have been put forward. Given the hypothesised small effect size, I don't find that the case for the significance of this project is compelling.

<u>Assessor 2</u>
03. Team quality and capability – relative to opportunity (Refer to the Team Quality and Capability section of the Grant proposal.
Assessors will:
- *Assess the track record of the CI team as a whole, relative to opportunity (Evaluate the support Junior Researchers (if applicable) have within the team), in context to the proposed research and time commitment of each CI.*
- *Consider all disclosed career disruptions and whether the referenced career disruption meets the NHMRC's definition (Section 6.1.1 of the NHMRC Funding Rules 2015) and if so, what the impact of the career disruption has had on the applicant's research achievements against all of the assessment criteria. Further advice pertaining to career disruptions can be found at Attachment H of the Project Grants Scheme Specific peer review guidelines.*
- *Evaluate clinical/teaching loads, output (publication quality and/or frequency) and contribution to discipline or field of expertise.*
- *Not consider journal impact factors or person centric metrics such as H-index. Citations are appropriate.)*

This research proposal has been put forward by a multidisciplinary group of researchers from Australia, with one highly experienced CI from the USA (Jacobsen). The team is comprised of individuals with extensive experience in conducting randomised controlled trials, the assessment of support needs amongst cancer populations, and the provision of psychosocial support for cancer patients. Adding strength to this team is CI O'Brien who has extensive experience working at the Cancer Council NSW at the operational level, and CI Boltong, who is based at Cancer Council Victoria. Statistical and research design advice will be provided by the very experienced CI McElduff. I applaud the researcher's inclusion of an ECR researcher (CI Boyes) who has demonstrated in a short space of time an excellent track record of publication and grant funding. Overall, this team is highly experienced and diverse, addressing the necessary skills to undertake the proposed research. In addition, several of the team members have previously collaborated, resulting in several well received publications in related areas.

<u>Assessor 2</u>
Budget Comments

Generally, the budget is appropriate for the scope of the proposed project. It is not particularly clear why the postdoc is required for 40% time only, nor how many telephone interviewers will be employed for the stated 50%, 100% and 50% requested budget. The requested budget for the database amendments is appropriate for this scope of work. Some more detail on the need for the visits to and from the Cancer Council offices would be helpful (assume that some of this is for the team meetings?).

<u>Assessor 2</u>
Overall Comments

Overall this is an interesting proposed project that has the capacity to improve support services for individuals with cancer, particularly for the thousands of cancer survivors. However, some limitations of the study design outlined previously detract from the overall proposal.

<u>Assessor 3</u>
01. Scientific Quality (Refer to the information available in the Research Proposal section of the Grant Proposal.
Assessors will:
- *Identify major and minor strengths and weaknesses in the proposed research plan.*
- *Consider the clarity and feasibility of the hypothesis or research objectives.*
- *Ask specific questions the Applicant can answer.*
- *Not provide a narrative or description of the proposed research.)*

This application has the potential to address an important issue in cancer care. However the researchers provide no pilot data to indicate the proposed methodology is acceptable to cancer council clients and feasible in the context of cancer council's models of care. Particular issues that may need to be addressed is the potential for contamination between consultants within the usual care and intervention groups given they sit in the same workspace. The number of screening questions may also be problematic both in terms of time to complete when a non-consented and distressed participant is asking for support with the potential for consultants to deviate from

protocol and/or impact on the quality of service delivery/uptake of services. Although I acknowledge that these factors will be assessed in the RCT preliminary data guiding the study design has not been provided and the methodology differs sufficiently from that used in QLD that there may be issues with acceptability. With respect to the primary outcome measure GHQ-28, it is not clear how baseline levels of distress will be controlled for given multiple distress measures are being used at b/l and 3/6months. Reliance on GHQ-28 at 3 months to control for individual differences at 6 months limits the ability to determine whether improvements were directly attributable to the structured intervention. The impact of the research could be strengthened by the inclusion of a more robust cost benefit analysis rather than subjective assessment using focus groups.

<u>Assessor 3</u>
02. Significance of the expected outcomes AND/OR Innovation of the concept (Refer to the information available in the Research Proposal section of the Grant Proposal.
Assessors will:
- *Consider whether the proposed research has potential to increase knowledge about human health, disease diagnoses, or biology of agents that affect human health. Consideration should also be applied to the application of new ideas, procedures, technologies, programs or health policy settings to important topics that will impact on human health.*
- *Assess any new/novel transformative aims and cutting edge techniques which could result in a paradigm shift affecting current practices or approaches.*
- *Reflect upon whether the proposed research is likely to result in yielding highly influential publications.*
- *Evaluate potential interest from other external parties, for example other researchers or community groups, following the outcomes of the research.*
- *Ask associated specific questions the Applicant can answer.*
- *Not provide a narrative or description of the proposed research.)*

The proposed research answers an important service delivery question for community-based cancer helpline organizations both locally and internationally. However relevance to the broader psychosocial context is less clear.

<u>Assessor 3</u>
03. Team quality and capability – relative to opportunity (Refer to the Team Quality and Capability section of the Grant proposal.
Assessors will:
- *Assess the track record of the CI team as a whole, relative to opportunity (Evaluate the support Junior Researchers (if applicable) have within the team), in context to the proposed research and time commitment of each CI.*
- *Consider all disclosed career disruptions and whether the referenced career disruption meets the NHMRC's definition (Section 6.1.1 of the NHMRC Funding Rules 2015) and if so, what the impact of the career disruption has had on the applicant's research achievements against all of the assessment criteria. Further advice pertaining to career disruptions can be found at Attachment H of the Project Grants Scheme Specific peer review guidelines.*
- *Evaluate clinical/teaching loads, output (publication quality and/or frequency) and contribution to discipline or field of expertise.*
- *Not consider journal impact factors or person centric metrics such as H-index. Citations are appropriate.)*

The team has the experience to deliver the proposed intervention. It is a good balance between experienced methodologists, leaders in psychosocial distress research, implementation and service delivery. the team would benefit from health economic expertise to better define how to achieve aim 2 of the research.Inclusion of a number of ECRs is appropriate and builds research capacity in psycho-oncology.

<u>Assessor 3</u>
Budget Comments

The budget is appropriate for the proposed work. Level of PSP appointments proposed are also appropriate for skill level required

<u>Assessor 3</u>
Overall Comments

This application addresses and important area of cancer care but the application may be premature given the lack of pilot data to support such a large RCT as proposed.

<u>Indigenous criteria comments (if applicable)</u>
